# Acute Osteomyelitis of the Symphysis Pubis after Inguinal Hernia Surgery

**DOI:** 10.1155/2015/845867

**Published:** 2015-04-20

**Authors:** Recep Tekin, Rojbin Ceylan Tekin, Figen Ceylan Cevik, Remzi Cevik

**Affiliations:** ^1^Department of Infectious Disease and Clinical Microbiology, Dicle University School of Medicine, Yenişehir, 21280 Diyarbakir, Turkey; ^2^Department of Radiology, Genesis Hospital, Diyarbakir, Turkey; ^3^Department of Physical Medicine and Rehabilitation, Diyarbakir State Hospital, Diyarbakir, Turkey; ^4^Department of Physical Medicine and Rehabilitation, Dicle University School of Medicine, Yenişehir, 21280 Diyarbakir, Turkey

## Abstract

Osteomyelitis of pubic symphysis is infectious inflammatory condition of the symphysis pubis and rare complication of surgery around inguinal and groin region. It should be kept in mind in the differential diagnosis of lower pelvic pain and should be sought in cases of pelvic insufficiency fractures. Herein, we present a case of a 55-year-old man with osteomyelitis of the symphysis pubis following inguinal hernia surgery for diagnosis and management of this rare condition.

## 1. Introduction

Osteomyelitis of the symphysis pubis is a rarely described entity. Osteomyelitis pubis is a term used to describe an entity characterised by severe pelvic pain, a wide-based gait, and bony destruction of the margins of the pubic symphysis [1]. Osteomyelitis of the symphysis pubis is very unusual and the clinical presentation can resemble osteitis pubis. Both entities commonly confused with each other. Rarity of condition nonspecific signs and symptoms has led to misdiagnosed, undiagnosed and delay in the management [2, 3]. Osteomyelitis of the symphysis pubis following inguinal hernia repair is extremely rare. We reported a rarely encountered case of osteomyelitis of the symphysis pubis.

## 2. Case

**Figure 1:**
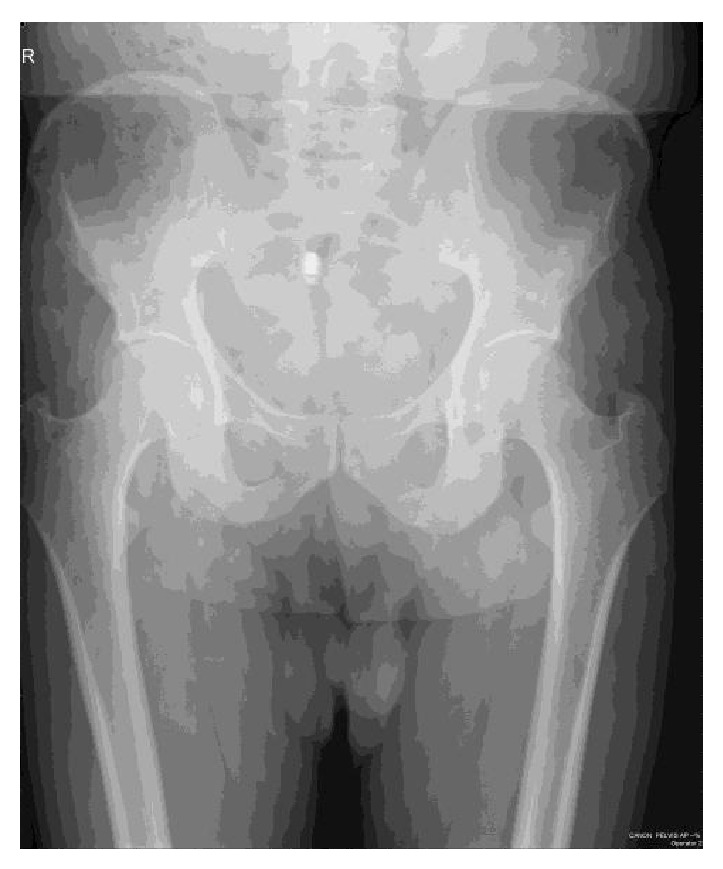
Anteroposterior plain radiograph of the pubis is normal.

**Figure 2:**
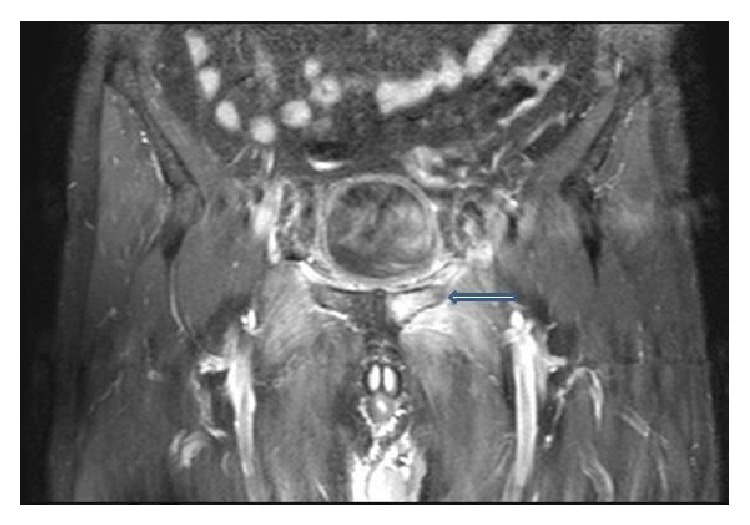
Pelvic MRI demonstrated inflammatory change in left pubic bone (arrow) and adductor muscle.

**Figure 3:**
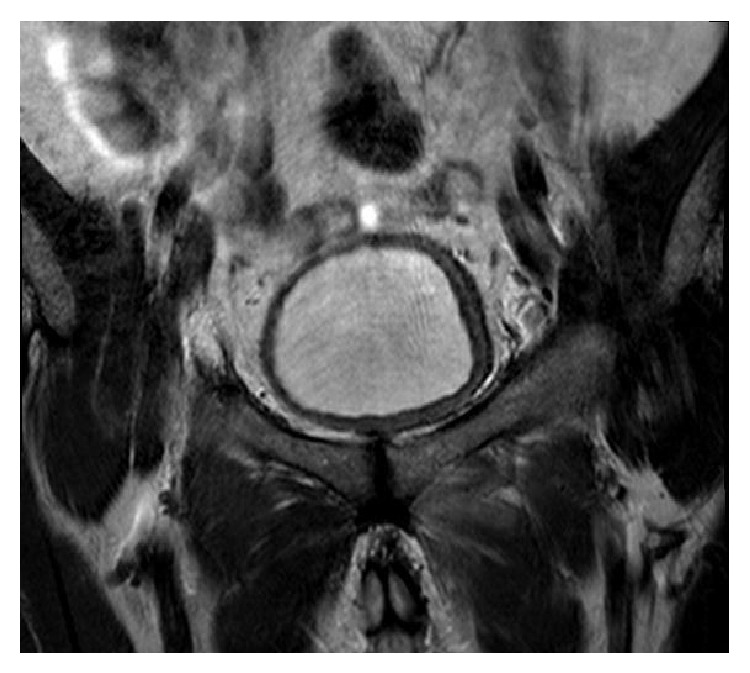
Pelvic MRI two months after completion of antibiotic therapy reveals no inflammatory change.

A 55-year-old man visited our hospital, because of fever and left inguinal pain that appeared at ten days after operation with disturbance of gait. On physical examination, his temperature was 39°C, pulse 102 beats/min, and blood pressure 120/80 mmHg. The locomotor system examination pelvic compression test and bitrochanteric compression test were positive. Investigation revealed white blood cell (WBC) count 14000 mm^3^, erythrocyte sedimentation rate 42 mm/h, and C-reactive protein (CRP) 9 mg/dL (normal range 0–5 mg/dL). The rheumatoid factor (RF), anti-nuclear antibody (ANA), HLAB-27, and standard tube agglutination for* Brucella* were negative. Anteroposterior plain radiograph of the pelvis is normal (Figure 1). Pelvic magnetic resonance imaging (MRI) demonstrated inflammatory change in left pubic bone, adductor muscle, which suggested the diagnosis of osteomyelitis of the pubis (Figure 2). The diagnosis of osteomyelitis of the symphysis pubis was based on review of the patient's history, clinical findings, and radiological abnormalities. Treatment was started with a combination of ampicillin/sulbactam 6 g/day and ciprofloxacin 1500 mg/day. After ten days of medical treatment, inguinal pain had reduced progressively. The therapy was stopped after 6 weeks; he was very healthy, inflammatory manifestation in left pubic bone in MRI regressed (Figure 3), and there was no recurrence of osteomyelitis of the symphysis pubis at one year after operation.

## 3. Discussion

Osteomyelitis of the symphysis pubis is a rarely described entity. The etiologies of pubic osteomyelitis include hematogenous, traumatic, and iatrogenic (after urological or gynecological surgery) factors, and the influence of other adjacent infection foci (e.g., prostate abscess, soft tissue infection) [3]. The pathogenesis is still not clear. Pubic osteomyelitis is an entity characterized by pelvic pain, wide-based gait, and bony destruction of the margins of the pubic symphysis. It is difficult clinically to identify infection of the pubis symphysis, as the symptoms are very nonspecific and do not present like a typical septic arthritis of a joint. However, we recommend having a high level of suspicion for patients presenting with pubic, groin, and hip pain, especially in patients who have undergone inguinal hernia surgery. Ross and Hu reviewed 100 cases of pubic symphysis septic arthritis [4]. They demonstrated that this condition is not specific to any age group and can range from 7 to 86 years of age. Most common presenting signs and symptoms include fever, pubic tenderness, antalgic gait, and pain with active/passive range of motion of hip. They identified several predisposing factors including urological, gynecological procedures, pregnancy, athletes, malignancy, and intravenous drug use, as well as other invasive procedures. Osteomyelitis of pubis should be considered in patients with pelvic pain, pubic tenderness, fever, and painful hip abduction. Pain specially occurred by gait and spread to the perineal, testicular, suprapubic, and inguinal area. However, pain also aggravated by Valsalva maneuver and occurred in scrotum after ejaculation [1]. C-reactive protein level, erythrocyte sedimentation rate, and white blood cell count are either elevated in osteomyelitis pubis, while they are usually normal or slightly elevated in osteitis pubis [5]. Osteomyelitis of the pubis frequently begins unilaterally and then expands to the other side of the pubic ramus. Symmetrical involvement is mostly present and bone destruction is progressive. Because radiographic imaging may be normal in acute pubic osteomyelitis, advanced imaging modalities should be considered in clinical suspicion. In the present case, radiographic examination was normal while inflammatory changes suggesting osteomyelitis were detected in MRI. Aspiration or open biopsy should be performed in the patients who do not respond to conservative treatment by means of clinical, laboratory, and radiological findings. However, most of the clinicians suggest taking direct biopsy without following conservative treatment response in patients who have inguinal pain increasing by gait and supporting laboratory and radiological findings after surgical procedure [1–3]. Because the present case did not accept biopsy, we diagnosed acute osteomyelitis pubis by clinically having fever and pelvic pain and elevations in CRP and WBC and demonstrating changes for osteomyelitis in MRI. The histological features of acute osteomyelitis pubis include granulation tissue, areas of necrosis (sequestra), exudates, lymphocytic infiltration, and plasma cells. In chronic osteomyelitis pubis similar features are noted, but in addition decreased vascularity and sclerotic new bone formation can be observed. [5]. In present case there was no need for the biopsy because of inguinal and pelvic pain increasing by gait, and laboratory and radiological findings supporting osteomyelitis of the symphysis pubis, and response to the conservative treatment.

Differential diagnosis of osteomyelitis of pubis should be made from tuberculosis, brucellosis, primer or metastatic bone neoplasms, pubic osteolysis, subluxation of symphysis pubis after delivery, muscle injury of pelvic girdle or sacroiliac joint, pelvic inflammatory disease, and ankylosing spondylitis. Histopathologic examination is an important diagnostic tool for differentiation from primer bone tumours rarely involving pubis such as multiple myeloma, reticular cell sarcoma, fibrosarcoma, and chondrosarcoma [3]. We excluded brucellosis by negative Rose Bengal test and inappropriate clinical and laboratory findings. In differential diagnosis, spondyloarthropathies should be taken into consideration. Because clinical and laboratory findings are absent, involved area is not usual place of enthesitis, and radiological finding is not resembled we excluded spondyloarthropathies including ankylosing spondylitis.

The treatment of osteomyelitis pubis is based on the intravenous administration of antibiotics. If the disease is progressive in spite of specific antibiotic therapy, surgical debridement with curettage and jet lavage is indicated. Invasive surgical procedures seem not to be necessary in most cases, due to the often spectacular response to simple antibiotherapy in arthritis and in discitis [6]. We recommend starting antibiotic immediately after culture is obtained for microbiology. Thus, it is important that these infections are identified early before the development of abscesses as surgical or radiological intervention might then be warranted. We opted for a treatment by high doses of ampicillin/sulbactam and ciprofloxacin for a duration of 6 weeks without surgical intervention, with a good clinical, laboratory, and radiologic response (Figure 3).

In conclusion, osteomyelitis of the pubic symphysis can have serious complications. The diagnosis should be considered in postoperative patients with disturbance of gait, particularly in elderly patients who have undergone an operation for inguinal hernia. It can be treated with antibiotic medication and early administration of it shall prevent developing of complication.
